# Redetermination of [Gd(NO_3_)_3_(H_2_O)_4_]·2H_2_O

**DOI:** 10.1107/S1600536812028000

**Published:** 2012-06-27

**Authors:** Ziyad A. Taha, Abdulaziz Ajlouni, Ahmed K. Hijazi, Fritz E. Kühn, Eberhardt Herdtweck

**Affiliations:** a Department of Applied Chemical Sciences, Jordan University of Science & Technology, Irbid 22110, Jordan; b Department of Chemistry, Faculty of Science, Jerash University, Jerash 26150, Jordan; c Department Chemie, Molekulare Katalyse, Technische Universität München, Lichtenbergstrasse 4, D-85747 Garching bei München, Germany; d Department Chemie, Lehrstuhl für Anorganische Chemie, Technische Universität München, Lichtenbergstrasse 4, D-85747 Garching bei München, Germany

## Abstract

The crystal structure of the title compound, tetra­aqua­tris­(nitrato-κ^2^ *O*,*O*′)gadolinium(III) dihydrate, was redetermined from single-crystal X-ray data. In comparison with the first determination [Ma *et al.* (1991 ▶). *Wuji Huaxue Xuebao*, **7**, 351–353], all H atoms could be located, accompanied with higher accuracy and precision. The Gd^III^ atom shows a ten-coordination with three nitrate ligands behaving in a bidentate manner and the other positions being occupied by four water mol­ecules, forming a distorted bicapped square anti­prism. Two nitrate ions coordinate to the metal atom with similar bond lengths while the third shows a more asymmetric bonding behaviour. An intricate network of O—H⋯O hydrogen bonds, including the lattice water mol­ecules, stabilizes the crystal packing.

## Related literature

For a previous determination of the title compound, see: Ma *et al.* (1991 ▶). Isotypic [*RE*(NO_3_)_3_(H_2_O)_4_]·2H_2_O structures were described for *RE* = Nd by Rogers *et al.* (1983 ▶), for Tb by Moret *et al.* (1990 ▶), for Sm by Kawashima *et al.* (2000 ▶), for Eu by Stumpf & Bolte (2001 ▶), for Dy by Gao *et al.* (1990 ▶), and for La by Eriksson *et al.* (1980 ▶). [*RE*(NO_3_)_3_(H_2_O)_4_]·H_2_O structures with one less water mol­ecule were described for *RE* = Eu by Ribár *et al.* (1986 ▶), for Gd by Stockhause & Meyer (1997 ▶), and for Yb by Junk *et al.* (1999 ▶).

## Experimental

### 

#### Crystal data

[Gd(NO_3_)_3_(H_2_O)_4_]·2H_2_O
*M* *_r_* = 451.38
Triclinic, 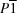

*a* = 6.6996 (2) Å
*b* = 9.1145 (3) Å
*c* = 11.6207 (3) Å
α = 69.8257 (10)°
β = 88.9290 (11)°
γ = 69.2170 (11)°
*V* = 618.36 (3) Å^3^
*Z* = 2
Mo *K*α radiation
μ = 5.45 mm^−1^
*T* = 173 K
0.48 × 0.46 × 0.23 mm

#### Data collection

Bruker APEXII CCD diffractometer
Absorption correction: multi-scan (*SADABS*; Bruker, 2008 ▶) *T*_min_ = 0.349, *T*_max_ = 0.745
19731 measured reflections
2256 independent reflections
2255 reflections with *I* > 2σ(*I*)
*R*_int_ = 0.036

#### Refinement

*R*[*F* ^2^ > 2σ(*F* ^2^)] = 0.016
*wR*(*F* ^2^) = 0.041
*S* = 1.20
2256 reflections
221 parameters
All H-atom parameters refined
Δρ_max_ = 1.04 e Å^−3^
Δρ_min_ = −1.10 e Å^−3^

### 

Data collection: *APEX2* (Bruker, 2008 ▶); cell refinement: *SAINT* (Bruker, 2008 ▶); data reduction: *SAINT*; program(s) used to solve structure: *SIR92* (Altomare *et al.*, 1994 ▶); program(s) used to refine structure: *SHELXL97* (Sheldrick, 2008 ▶); molecular graphics: *PLATON* (Spek, 2009 ▶); software used to prepare material for publication: *PLATON*.

## Supplementary Material

Crystal structure: contains datablock(s) global, I. DOI: 10.1107/S1600536812028000/wm2634sup1.cif

Structure factors: contains datablock(s) I. DOI: 10.1107/S1600536812028000/wm2634Isup2.hkl

Additional supplementary materials:  crystallographic information; 3D view; checkCIF report

## Figures and Tables

**Table 1:** Selected bond lengths (Å)

Gd1—O1	2.528 (2)
Gd1—O2	2.494 (3)
Gd1—O4	2.578 (2)
Gd1—O5	2.518 (3)
Gd1—O7	2.552 (2)
Gd1—O8	2.754 (2)
Gd1—O10	2.398 (2)
Gd1—O11	2.389 (2)
Gd1—O12	2.392 (3)
Gd1—O13	2.364 (3)

**Table 2:** Hydrogen-bond geometry (Å, °)

*D*—H⋯*A*	*D*—H	H⋯*A*	*D*⋯*A*	*D*—H⋯*A*
O10—H1⋯O15^i^	0.76 (5)	1.97 (5)	2.720 (4)	171 (5)
O10—H2⋯O4^ii^	0.75 (4)	2.21 (4)	2.956 (3)	178 (6)
O11—H3⋯O14^iii^	0.82 (4)	1.85 (4)	2.671 (3)	176 (5)
O11—H4⋯O7^iv^	0.78 (5)	2.19 (5)	2.967 (4)	173 (5)
O12—H5⋯O4^v^	0.77 (6)	2.50 (6)	3.185 (3)	149 (5)
O12—H5⋯O7^v^	0.77 (6)	2.56 (6)	3.156 (4)	135 (5)
O12—H6⋯O8^vi^	0.83 (5)	2.27 (5)	3.074 (3)	162 (4)
O12—H6⋯O9^vi^	0.83 (5)	2.39 (5)	3.062 (4)	139 (4)
O13—H7⋯O15	0.90 (4)	1.84 (4)	2.721 (3)	169 (5)
O13—H8⋯O14	0.71 (4)	2.04 (4)	2.738 (4)	168 (5)
O14—H9⋯O9^iv^	0.81 (5)	2.03 (5)	2.826 (4)	167 (4)
O14—H10⋯O3^v^	0.76 (5)	2.45 (5)	3.008 (4)	132 (5)
O14—H10⋯O5^vii^	0.76 (5)	2.31 (5)	2.888 (4)	134 (5)
O15—H11⋯O6^ii^	0.80 (6)	2.02 (6)	2.819 (4)	176 (6)
O15—H12⋯O3^viii^	0.76 (6)	2.30 (6)	2.903 (4)	139 (5)

Symmetry codes: (i) 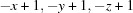; (ii) 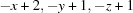; (iii) 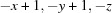; (iv) 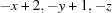; (v) 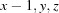; (vi) 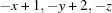; (vii) 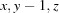; (viii) 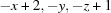.

**Fig. 1.**
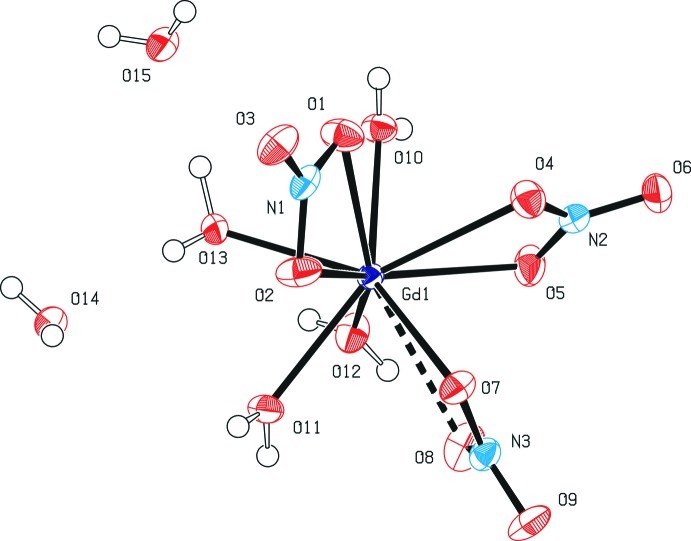
View of the title compound with the atom numbering scheme. Displacement ellipsoids for non-H atoms are drawn at the 50% probability level.

**Fig. 2.**
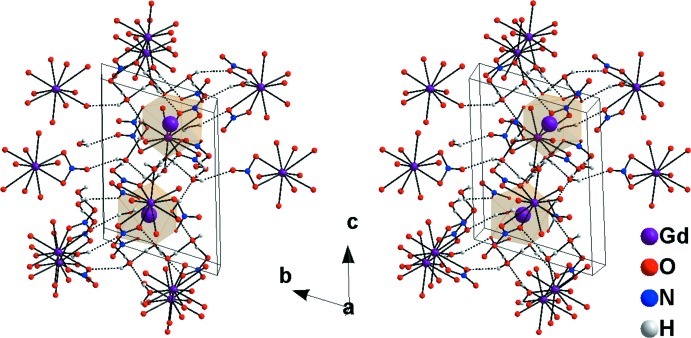
Stereo plot of the expanded unit cell. Hydrogen bonds (dotted lines) are limited to a distance O···H of 2.35 Å.

## supplementary crystallographic information

### Comment

Single crystals of gadolinium nitrate hexahydrate, [Gd(NO_3_)_3_(H_2_O)_4_]^.^2H_2_O, were obtained and its structure was re-determined using single-crystal X-ray diffraction analysis.

As shown in Figure 1, the Gd^III^ atom is ten-coordinated, being bound to six nitrate-oxygen atoms, O1, O2, O4, O5, O7, and O8, and four water-oxygen atoms O10, O11, O12, and O13. The nitrate ions are coordinated to the central gadolinium atom in a bidentate mode and show clearly the changes in bond lengths and angles noted previously for the isotypic Eu(III) structure (Stumpf & Bolte, 2001). The distances between the Gd^III^ ion and the nitrate-O atoms (Gd—O) are in the range 2.494 (3)–2.754 (2) Å, with a mean value of 2.571 Å. The differences between the Gd^III^ atom and the two oxygen atoms of the same nitrate group are 0.034 Å and 0.060 Å for nitrate N1 and nitrate N2, respectively. Nitrate N1 and nitrate N2 groups appear to be more symmetrically bonded to the Gd^III^ atom because the third nitrate group N3 exhibits one Gd—O distance that is 0.202 Å longer than the other. This asymmetric bonding seem to be associated with a steric effect of the coordinating water molecules. There is simply not space enough for all the oxygen atoms around the Gd^III^ atom at the same distance. The Gd—O_water_ distances are in the range of 2.364 (3)–2.398 (2) Å with an average distance of 2.386 Å. Hence the water molecules are closer to the Gd^III^ atom than the nitrate groups by *ca.* 0.185 Å.

These results are comparable to that reported for other lanthanide complexes with bidentately coordinating nitrate groups. The coordination polyhedron around the Gd^III^ ion can be best described as a distorted bi-capped square antiprism (Fig. 1), as previously reported for other hydrated lanthanide(III) nitrate complexes (Ribár *et al.*, 1986; Moret *et al.*, 1990; Ma *et al.*, 1991, Stockhause & Meyer, 1997; Kawashima *et al.*, 2000; Stumpf & Bolte, 2001; Junk *et al.*, 1999; Gao *et al.*, 1990; Eriksson *et al.*, 1980; Rogers *et al.*, 1983). The structure contains additional two lattice water molecules, which are associated with the complex by a network of hydrogen bonds. All hydrogen atoms and except for O1 and O2 all oxygen atoms are involved in a complicated network of O—H···O hydrogen bonds, stabilizing the crystal packing (Table 1, Fig. 2). H5, H6 and H10 act as bifurcated bridging atoms. In contrast to the original work of Ma *et al.* (1991), we report much more precise results. All hydrogen atoms could be located in the difference Fourier maps and were allowed to refine freely. The final *R*1 value changed from 0.076 to 0.0158 and the overall e.s.d.'s for the positional parameters dropped from *e.g.* Gd1 *x* = 0.8016 (1) to 0.80352 (2). The same is valid for distances *e.g.* Gd1—O1 dropped from 2.558 (56) Å to 2.528 (2) Å and for the angels *e.g.* O1—Gd1—O2 dropped from 51.1 (22) to 50.56 (7).

### Experimental

Single crystals of [Gd(NO_3_)_3_(H_2_O)_4_]^.^2H_2_O have been obtained accidentally during the synthesis of a Gd—*N*,*N*-bis(salicylicaldehyde)-*o*-phenylenediamine Schiff base complex. 1.0 mmol of the Schiff base were dissolved in 10 ml chloroform. To this solution was added in a drop-wise manner a 10 ml ethyl acetate solution of 2.0 mmol Gd(NO_3_)_3_^.^6(H_2_O). The reaction mixture was stirred for 2 h at room temperature. The yellow precipitate was filtered, washed several times with ethyl acetate and chloroform. Single crystals suitable for X-ray were obtained after a few days by the slow evaporation of the solvent in an open atmosphere.

### Refinement

Hydrogen atoms could be located in difference Fourier maps and were allowed to refine freely. The residual electron density of Δρmax = 1.04 e Å^-3^ is located 0.89 Å next to Gd1, whereas Δρmin = 1.10 e Å^-3^ is located 0.96 Å next to Gd1.

### Crystal data

**Table:**

[Gd(NO_3_)_3_(H_2_O)_4_]·2H_2_O	*Z* = 2
*M**_r_* = 451.38	*F*(000) = 434
Triclinic, *P*1	*D*_x_ = 2.424 Mg m^−^^3^
Hall symbol: -P 1	Mo *K*α radiation, λ = 0.71073 Å
*a* = 6.6996 (2) Å	Cell parameters from 9802 reflections
*b* = 9.1145 (3) Å	θ = 1.9–25.4°
*c* = 11.6207 (3) Å	µ = 5.45 mm^−^^1^
α = 69.8257 (10)°	*T* = 173 K
β = 88.9290 (11)°	Fragment, orange
γ = 69.2170 (11)°	0.48 × 0.46 × 0.23 mm
*V* = 618.36 (3) Å^3^	

### Data collection

**Table:**

Bruker APEXII CCD diffractometer	2256 independent reflections
Radiation source: rotating anode FR591	2255 reflections with *I* > 2σ(*I*)
Graphite monochromator	*R*_int_ = 0.036
Detector resolution: 16 pixels mm^-1^	θ_max_ = 25.4°, θ_min_ = 1.9°
phi– and ω–rotation scans	*h* = −8→8
Absorption correction: multi-scan (*SADABS*; Bruker, 2008)	*k* = −10→10
*T*_min_ = 0.349, *T*_max_ = 0.745	*l* = −13→13
19731 measured reflections	

### Refinement

**Table:**

Refinement on *F*^2^	Secondary atom site location: difference Fourier map
Least-squares matrix: full	Hydrogen site location: difference Fourier map
*R*[*F*^2^ > 2σ(*F*^2^)] = 0.016	All H-atom parameters refined
*wR*(*F*^2^) = 0.041	Weighting scheme based on measured s.u.'s *w* = 1/[σ^2^(*F*_o_^2^) + (0.0186*P*)^2^ + 0.725*P*] where *P* = (*F*_o_^2^ + 2*F*_c_^2^)/3
*S* = 1.20	(Δ/σ)_max_ = 0.001
2256 reflections	Δρ_max_ = 1.04 e Å^−^^3^
221 parameters	Δρ_min_ = −1.10 e Å^−^^3^
0 restraints	Extinction correction: *SHELXL97* (Sheldrick, 2008), Fc^*^=kFc[1+0.001xFc^2^λ^3^/sin(2θ)]^-1/4^
Primary atom site location: structure-invariant direct methods	Extinction coefficient: 0.0129 (7)

### Special details

**Table:**

Experimental. Diffractometer operator E. Herdtweck scanspeed 10 s per frame dx 45 4932 frames measured in 9 data sets phi-scan with delta_phi = 0.50 omega-scans with delta_omega = 0.50
Geometry. Bond distances, angles *etc*. have been calculated using the rounded fractional coordinates. All su's are estimated from the variances of the (full) variance-covariance matrix. The cell e.s.d.'s are taken into account in the estimation of distances, angles and torsion angles
Refinement. Refinement on *F*^2^ for ALL reflections except for 0 with very negative *F*^2^ or flagged by the user for potential systematic errors. Weighted *R*-factors *wR* and all goodnesses of fit *S* are based on *F*^2^, conventional *R*-factors *R* are based on *F*, with *F* set to zero for negative *F*^2^. The observed criterion of *F*^2^ > σ(*F*^2^) is used only for calculating R_factor_obs *etc*. and is not relevant to the choice of reflections for refinement. *R*-factors based on *F*^2^ are statistically about twice as large as those based on *F*, and *R*- factors based on ALL data will be even larger.

### Fractional atomic coordinates and isotropic or equivalent isotropic displacement parameters (Å^2^)

**Table:**

	*x*	*y*	*z*	*U*_iso_*/*U*_eq_	
Gd1	0.80352 (2)	0.59425 (1)	0.22568 (1)	0.0147 (1)	
O1	1.0817 (4)	0.3431 (3)	0.3878 (2)	0.0294 (7)	
O2	1.1029 (4)	0.3531 (3)	0.20104 (19)	0.0286 (6)	
O3	1.3338 (4)	0.1425 (3)	0.3490 (2)	0.0304 (6)	
O4	1.0578 (3)	0.6801 (3)	0.3296 (2)	0.0268 (6)	
O5	0.7353 (4)	0.8617 (3)	0.2653 (2)	0.0305 (7)	
O6	0.9570 (4)	0.9177 (3)	0.3616 (2)	0.0319 (7)	
O7	1.0495 (3)	0.6937 (3)	0.07347 (19)	0.0245 (6)	
O8	0.7274 (4)	0.8794 (3)	0.0190 (2)	0.0336 (7)	
O9	0.9806 (4)	0.9195 (3)	−0.0904 (2)	0.0322 (7)	
O10	0.6976 (4)	0.5590 (3)	0.4287 (2)	0.0224 (7)	
O11	0.7203 (4)	0.5521 (3)	0.04330 (19)	0.0223 (6)	
O12	0.4279 (4)	0.7621 (3)	0.1754 (2)	0.0259 (7)	
O13	0.6339 (4)	0.3963 (3)	0.2792 (2)	0.0262 (7)	
N1	1.1767 (4)	0.2751 (3)	0.3141 (2)	0.0213 (7)	
N2	0.9171 (4)	0.8234 (3)	0.3202 (2)	0.0233 (8)	
N3	0.9183 (4)	0.8342 (3)	−0.0015 (2)	0.0212 (7)	
O14	0.5912 (4)	0.2215 (3)	0.1372 (2)	0.0239 (7)	
O15	0.6255 (4)	0.2025 (3)	0.5159 (2)	0.0258 (7)	
H1	0.608 (7)	0.632 (6)	0.437 (4)	0.038 (12)*	
H2	0.760 (6)	0.500 (5)	0.490 (4)	0.028 (11)*	
H3	0.625 (6)	0.625 (5)	−0.011 (4)	0.025 (10)*	
H4	0.789 (7)	0.491 (6)	0.012 (4)	0.037 (12)*	
H5	0.341 (8)	0.724 (6)	0.193 (5)	0.056 (15)*	
H6	0.369 (7)	0.865 (6)	0.135 (4)	0.046 (12)*	
H7	0.649 (7)	0.328 (6)	0.358 (4)	0.043 (11)*	
H8	0.640 (6)	0.344 (5)	0.245 (3)	0.019 (10)*	
H9	0.705 (7)	0.183 (5)	0.113 (4)	0.035 (11)*	
H10	0.561 (7)	0.151 (6)	0.181 (4)	0.048 (14)*	
H11	0.741 (9)	0.167 (6)	0.554 (5)	0.053 (15)*	
H12	0.589 (9)	0.131 (7)	0.522 (5)	0.069 (18)*	

### Atomic displacement parameters (Å^2^)

**Table:**

	*U*^11^	*U*^22^	*U*^33^	*U*^12^	*U*^13^	*U*^23^
Gd1	0.0156 (1)	0.0131 (1)	0.0141 (1)	−0.0049 (1)	0.0013 (1)	−0.0038 (1)
O1	0.0320 (12)	0.0258 (11)	0.0220 (11)	0.0000 (9)	−0.0012 (9)	−0.0096 (9)
O2	0.0307 (12)	0.0234 (11)	0.0190 (10)	0.0007 (9)	0.0036 (9)	−0.0040 (9)
O3	0.0254 (11)	0.0149 (10)	0.0366 (12)	0.0005 (9)	0.0011 (9)	−0.0006 (9)
O4	0.0246 (11)	0.0262 (11)	0.0298 (11)	−0.0082 (9)	0.0003 (9)	−0.0116 (9)
O5	0.0296 (12)	0.0181 (10)	0.0389 (13)	−0.0076 (9)	−0.0101 (10)	−0.0053 (9)
O6	0.0422 (14)	0.0241 (12)	0.0326 (12)	−0.0157 (11)	−0.0049 (10)	−0.0102 (10)
O7	0.0239 (11)	0.0200 (11)	0.0241 (10)	−0.0073 (9)	0.0020 (8)	−0.0024 (8)
O8	0.0213 (11)	0.0266 (12)	0.0495 (14)	−0.0051 (9)	0.0113 (10)	−0.0140 (11)
O9	0.0416 (13)	0.0205 (11)	0.0261 (11)	−0.0107 (10)	0.0118 (10)	0.0002 (9)
O10	0.0258 (12)	0.0208 (12)	0.0175 (11)	−0.0050 (10)	0.0025 (9)	−0.0072 (10)
O11	0.0260 (11)	0.0204 (11)	0.0162 (10)	−0.0040 (9)	−0.0005 (9)	−0.0061 (9)
O12	0.0182 (11)	0.0195 (12)	0.0354 (12)	−0.0055 (9)	−0.0007 (9)	−0.0058 (10)
O13	0.0445 (14)	0.0267 (12)	0.0178 (11)	−0.0233 (11)	0.0068 (9)	−0.0101 (10)
N1	0.0203 (12)	0.0136 (12)	0.0264 (13)	−0.0063 (10)	0.0027 (10)	−0.0029 (10)
N2	0.0305 (14)	0.0202 (13)	0.0182 (12)	−0.0125 (11)	−0.0018 (10)	−0.0023 (10)
N3	0.0247 (13)	0.0162 (12)	0.0222 (12)	−0.0070 (10)	0.0054 (10)	−0.0070 (10)
O14	0.0277 (12)	0.0189 (11)	0.0232 (11)	−0.0083 (10)	0.0008 (9)	−0.0057 (9)
O15	0.0328 (14)	0.0201 (12)	0.0245 (11)	−0.0118 (10)	0.0039 (10)	−0.0061 (9)

### Geometric parameters (Å, º)

**Table:**

Gd1—O1	2.528 (2)	O7—N3	1.277 (3)
Gd1—O2	2.494 (3)	O8—N3	1.243 (4)
Gd1—O4	2.578 (2)	O9—N3	1.227 (3)
Gd1—O5	2.518 (3)	O10—H1	0.76 (5)
Gd1—O7	2.552 (2)	O10—H2	0.75 (4)
Gd1—O8	2.754 (2)	O11—H3	0.82 (4)
Gd1—O10	2.398 (2)	O11—H4	0.78 (5)
Gd1—O11	2.389 (2)	O12—H5	0.77 (6)
Gd1—O12	2.392 (3)	O12—H6	0.83 (5)
Gd1—O13	2.364 (3)	O13—H7	0.90 (4)
O1—N1	1.260 (4)	O13—H8	0.71 (4)
O2—N1	1.268 (3)	O14—H9	0.81 (5)
O3—N1	1.228 (4)	O14—H10	0.76 (5)
O4—N2	1.280 (4)	O15—H11	0.80 (6)
O5—N2	1.259 (4)	O15—H12	0.76 (6)
O6—N2	1.223 (4)		
			
O1—Gd1—O2	50.56 (7)	O8—Gd1—O12	68.70 (8)
O1—Gd1—O4	67.93 (8)	O8—Gd1—O13	130.50 (8)
O1—Gd1—O5	111.77 (8)	O10—Gd1—O11	140.10 (9)
O1—Gd1—O7	99.76 (8)	O10—Gd1—O12	79.72 (8)
O1—Gd1—O8	146.55 (9)	O10—Gd1—O13	71.04 (9)
O1—Gd1—O10	68.65 (9)	O11—Gd1—O12	78.30 (9)
O1—Gd1—O11	115.74 (9)	O11—Gd1—O13	71.47 (8)
O1—Gd1—O12	143.98 (8)	O12—Gd1—O13	75.61 (9)
O1—Gd1—O13	78.10 (9)	Gd1—O1—N1	95.96 (16)
O2—Gd1—O4	93.36 (8)	Gd1—O2—N1	97.41 (18)
O2—Gd1—O5	141.08 (9)	Gd1—O4—N2	95.25 (17)
O2—Gd1—O7	68.28 (8)	Gd1—O5—N2	98.75 (19)
O2—Gd1—O8	109.78 (8)	Gd1—O7—N3	101.83 (17)
O2—Gd1—O10	117.19 (8)	Gd1—O8—N3	92.94 (17)
O2—Gd1—O11	69.75 (8)	Gd1—O10—H2	129 (3)
O2—Gd1—O12	145.36 (9)	H1—O10—H2	110 (5)
O2—Gd1—O13	81.74 (9)	Gd1—O10—H1	118 (3)
O4—Gd1—O5	49.97 (8)	H3—O11—H4	105 (5)
O4—Gd1—O7	69.84 (7)	Gd1—O11—H3	122 (3)
O4—Gd1—O8	89.68 (8)	Gd1—O11—H4	131 (4)
O4—Gd1—O10	75.39 (8)	Gd1—O12—H6	128 (3)
O4—Gd1—O11	144.41 (8)	Gd1—O12—H5	122 (4)
O4—Gd1—O12	120.88 (9)	H5—O12—H6	109 (5)
O4—Gd1—O13	138.89 (7)	Gd1—O13—H8	122 (3)
O5—Gd1—O7	84.58 (8)	Gd1—O13—H7	120 (3)
O5—Gd1—O8	64.27 (7)	H7—O13—H8	104 (5)
O5—Gd1—O10	70.94 (8)	H9—O14—H10	110 (5)
O5—Gd1—O11	130.51 (8)	H11—O15—H12	110 (6)
O5—Gd1—O12	71.40 (9)	O1—N1—O2	116.1 (3)
O5—Gd1—O13	133.03 (9)	O1—N1—O3	122.2 (2)
O7—Gd1—O8	47.73 (8)	O2—N1—O3	121.7 (3)
O7—Gd1—O10	145.14 (8)	O4—N2—O5	116.0 (3)
O7—Gd1—O11	74.76 (8)	O4—N2—O6	122.0 (3)
O7—Gd1—O12	116.18 (8)	O5—N2—O6	122.0 (3)
O7—Gd1—O13	140.84 (8)	O8—N3—O9	122.0 (3)
O8—Gd1—O10	131.16 (8)	O7—N3—O8	117.5 (2)
O8—Gd1—O11	68.65 (8)	O7—N3—O9	120.5 (3)
			
O2—Gd1—O1—N1	−0.36 (17)	O11—Gd1—O5—N2	−134.37 (16)
O4—Gd1—O1—N1	−114.9 (2)	O12—Gd1—O5—N2	170.31 (18)
O5—Gd1—O1—N1	−139.49 (18)	O13—Gd1—O5—N2	122.60 (17)
O7—Gd1—O1—N1	−51.5 (2)	O1—Gd1—O7—N3	−171.29 (17)
O8—Gd1—O1—N1	−63.6 (3)	O2—Gd1—O7—N3	148.39 (19)
O10—Gd1—O1—N1	162.8 (2)	O4—Gd1—O7—N3	−109.28 (19)
O11—Gd1—O1—N1	26.2 (2)	O5—Gd1—O7—N3	−60.09 (18)
O12—Gd1—O1—N1	132.50 (19)	O8—Gd1—O7—N3	−0.29 (16)
O13—Gd1—O1—N1	88.7 (2)	O10—Gd1—O7—N3	−104.8 (2)
O1—Gd1—O2—N1	0.36 (17)	O11—Gd1—O7—N3	74.51 (18)
O4—Gd1—O2—N1	57.96 (19)	O12—Gd1—O7—N3	6.1 (2)
O5—Gd1—O2—N1	75.6 (2)	O13—Gd1—O7—N3	105.6 (2)
O7—Gd1—O2—N1	124.7 (2)	O1—Gd1—O8—N3	16.5 (3)
O8—Gd1—O2—N1	148.82 (18)	O2—Gd1—O8—N3	−30.59 (19)
O10—Gd1—O2—N1	−17.3 (2)	O4—Gd1—O8—N3	62.87 (18)
O11—Gd1—O2—N1	−154.2 (2)	O5—Gd1—O8—N3	107.53 (19)
O12—Gd1—O2—N1	−130.31 (19)	O7—Gd1—O8—N3	0.29 (16)
O13—Gd1—O2—N1	−80.99 (19)	O10—Gd1—O8—N3	132.99 (18)
O1—Gd1—O4—N2	−148.15 (17)	O11—Gd1—O8—N3	−88.21 (18)
O2—Gd1—O4—N2	167.14 (15)	O12—Gd1—O8—N3	−173.6 (2)
O5—Gd1—O4—N2	1.56 (14)	O13—Gd1—O8—N3	−126.70 (18)
O7—Gd1—O4—N2	101.75 (16)	Gd1—O1—N1—O2	0.6 (3)
O8—Gd1—O4—N2	57.34 (16)	Gd1—O1—N1—O3	179.5 (3)
O10—Gd1—O4—N2	−75.63 (16)	Gd1—O2—N1—O1	−0.6 (3)
O11—Gd1—O4—N2	108.04 (19)	Gd1—O2—N1—O3	−179.5 (3)
O12—Gd1—O4—N2	−7.40 (18)	Gd1—O4—N2—O5	−2.7 (2)
O13—Gd1—O4—N2	−111.58 (18)	Gd1—O4—N2—O6	177.6 (2)
O1—Gd1—O5—N2	28.63 (19)	Gd1—O5—N2—O4	2.7 (2)
O2—Gd1—O5—N2	−24.9 (2)	Gd1—O5—N2—O6	−177.5 (2)
O4—Gd1—O5—N2	−1.59 (14)	Gd1—O7—N3—O8	0.5 (3)
O7—Gd1—O5—N2	−69.73 (17)	Gd1—O7—N3—O9	−179.1 (2)
O8—Gd1—O5—N2	−114.96 (19)	Gd1—O8—N3—O7	−0.5 (3)
O10—Gd1—O5—N2	85.07 (17)	Gd1—O8—N3—O9	179.1 (3)

### Hydrogen-bond geometry (Å, º)

**Table:**

*D*—H···*A*	*D*—H	H···*A*	*D*···*A*	*D*—H···*A*
O10—H1···O15^i^	0.76 (5)	1.97 (5)	2.720 (4)	171 (5)
O10—H2···O4^ii^	0.75 (4)	2.21 (4)	2.956 (3)	178 (6)
O11—H3···O14^iii^	0.82 (4)	1.85 (4)	2.671 (3)	176 (5)
O11—H4···O7^iv^	0.78 (5)	2.19 (5)	2.967 (4)	173 (5)
O12—H5···O4^v^	0.77 (6)	2.50 (6)	3.185 (3)	149 (5)
O12—H5···O7^v^	0.77 (6)	2.56 (6)	3.156 (4)	135 (5)
O12—H6···O8^vi^	0.83 (5)	2.27 (5)	3.074 (3)	162 (4)
O12—H6···O9^vi^	0.83 (5)	2.39 (5)	3.062 (4)	139 (4)
O13—H7···O15	0.90 (4)	1.84 (4)	2.721 (3)	169 (5)
O13—H8···O14	0.71 (4)	2.04 (4)	2.738 (4)	168 (5)
O14—H9···O9^iv^	0.81 (5)	2.03 (5)	2.826 (4)	167 (4)
O14—H10···O3^v^	0.76 (5)	2.45 (5)	3.008 (4)	132 (5)
O14—H10···O5^vii^	0.76 (5)	2.31 (5)	2.888 (4)	134 (5)
O15—H11···O6^ii^	0.80 (6)	2.02 (6)	2.819 (4)	176 (6)
O15—H12···O3^viii^	0.76 (6)	2.30 (6)	2.903 (4)	139 (5)

Symmetry codes: (i) −*x*+1, −*y*+1, −*z*+1; (ii) −*x*+2, −*y*+1, −*z*+1; (iii) −*x*+1, −*y*+1, −*z*; (iv) −*x*+2, −*y*+1, −*z*; (v) *x*−1, *y*, *z*; (vi) −*x*+1, −*y*+2, −*z*; (vii) *x*, *y*−1, *z*; (viii) −*x*+2, −*y*, −*z*+1.
